# High-Temperature Short-Time Preserves Human Milk's Bioactive Proteins and Their Function Better Than Pasteurization Techniques With Long Processing Times

**DOI:** 10.3389/fped.2021.798609

**Published:** 2022-01-20

**Authors:** Eva Kontopodi, Sjef Boeren, Bernd Stahl, Johannes B. van Goudoever, Ruurd M. van Elburg, Kasper Hettinga

**Affiliations:** ^1^Emma Children's Hospital, Amsterdam UMC, Amsterdam, Netherlands; ^2^Food Quality and Design Group, Wageningen University and Research, Wageningen, Netherlands; ^3^Laboratory of Biochemistry, Wageningen University, Wageningen, Netherlands; ^4^Department of Chemical Biology and Drug Discovery, Utrecht Institute for Pharmaceutical Sciences, Utrecht University, Utrecht, Netherlands; ^5^Danone Nutricia Research, Utrecht, Netherlands

**Keywords:** protein functionality, donor human milk, milk processing, holder pasteurization, HTST

## Abstract

Donor human milk is generally processed by holder pasteurization (HoP) at 62. 5°C for 30 min. This temperature-time combination is sufficient for eliminating pathogens in donor milk, but also negatively affects several bioactive milk components. Long heating up times may further affect the bioactive properties of pasteurized milk. High-Temperature-Short-Time (HTST), a treatment with shorter processing times (72°C for 15 sec), was investigated as a suitable alternative to HoP. In addition, pasteurization methods that follow the same temperature regime but with varying heating up times were compared. Human milk samples from four different donors were combined into one pool, which was then used to perform all analyses. The effects of these methods on the levels and functionality of immunoglobulin A, lactoferrin, lysozyme and bile salt-stimulated lipase, were evaluated with LC-MS/MS-based proteomics and activity assays, while the pasteurization efficacy was evaluated with an alkaline phosphatase test. HoP, a treatment with long processing times, times, caused the highest reduction in all proteins studied (reduced by 50–98%). Compounds such as lactoferrin and bile salt-stimulated lipase that are more sensitive to heat treatments were better retained with HTST, but their levels and functionality were still significantly lower than those of untreated donor milk (52 and 81% reduction of lactoferrin and bile salt-stimulated lipase activity, respectively). Our findings showed that a treatment with considerably shorter processing times, such as HTST, may reduce the thermal damage caused to the bioactive proteins compared to HoP, without affecting pasteurization efficacy. Since the vast majority of the donor human milk banks that are currently operating on a global level apply HoP to donor milk, our findings may provide relevant information for the optimization of donor milk processing.

## Introduction

Tailored to each infant's need, mother's own milk represents the optimal source of neonatal nutrition. The unique composition of human milk (HM) promotes healthy infant development and growth, while the numerous bioactive factors it contains, protect infants against infections and various diseases (1, 2). HM has been shown for example to be bacteriostatic against a number or bacteria, which is mainly attributed to the functionality of immunoglobulin A (IgA), lactoferrin (LTF) and lysozyme (LYZ). LTF, amongst others, limits the availability of the iron required for the growth of iron-dependent pathogens, LYZ disrupts cell walls in gram-positive bacteria, and secretory IgA is generally considered as the main antibody system in HM. Synergistic effects of these proteins have also been reported (3–5). Especially for premature infants, when mother's own milk is not available, donor human milk (DHM) represents the best alternative form of nutrition. Current evidence suggests that DHM protects against necrotizing enterocolitis when compared to infant formula, while its provision is correlated with improved long-term outcomes in preterm infants (2, 6). DHM should be provided though established human milk banks (HMBs) that can ensure its safety (7).

Before provision, HMBs subject DHM to holder pasteurization (HoP), a low-temperature long-time heat treatment at 62.5°C for 30 min (8). HoP effectively inactivates potential viral and bacterial agents, and it is known as the method recommended for DHM treatment in all international human milk banking guidelines (8, 9). However, this method has been shown to cause substantial losses in various bioactive milk components, due to the thermally induced denaturation occurring in these components (8, 10). In fact, as previously reviewed, losses from 20 to 90% in the levels and functionality of immunoglobulin A (IgA), lactoferrin (LTF), and lysozyme (LYZ) have been reported after this treatment (10). In addition, HoP results in the total inactivation of bile salt stimulated lipase (BSSL), a heat-labile enzyme involved in fat absorption and infant metabolism (10). The reported lower growth rates in preterm infants fed with DHM that underwent HoP, may be the result of this detrimental effect (11).

Different devices are currently being employed by HMBs to perform HoP. Standard pasteurizers are most commonly used, with water as the heating medium (8, 12). The ideal HoP process should be comprised of a rapid heating up phase to 62.5°C, a phase of constant temperature (30 min) and finally a rapid cooling down phase to <10°C (8, 9). A recent study investigating human milk banking practices in Europe, revealed that in contrast to the recommendations on pasteurization performance, DHM is currently exposed to long processing times (12). This could result in higher losses of DHM components. More specifically, losses of 1.6, 1.7, and 2.4% were documented for IgA, LYZ and LTF, respectively, for every minute spent at 62.5°C (13). Therefore, since the duration of the heat treatment and the temperature at which DHM is exposed highly affects the preservation of its bioactive components, ensuring short heating up times seems essential when using thermal techniques.

Various studies report that high-temperature-short-time (HTST) treatment may be a suitable alternative to HoP, as it was found to provide similar microbial reduction (e.g., in *E.coli, S.aureus, S. epidermidis, E. faecalis, P. aeruginosa, L. monocytogenes, S. agalactiae* and *C. sakazakii* counts) and to better retain the DHM bioactive proteins (14–19). HTST is usually performed by heating the milk at 72°C for a duration of 15 sec. This method is well-established in the dairy industry, and it usually involves the rapid heating of a thin layer of milk in a continuous flow system (20). The shorter treatment time as well as the shorter exposure time at the processing temperature may be the reason of the promising results reported after this treatment (8). However, substantial information on whether shorter heating up times during pasteurization can positively influence the retention of the DHM bioactive components, is at present lacking.

Our aim was to determine 1. the effect of shorter heating up times and 2. the effect of shorter pasteurization duration at a higher temperature on the preservation of DHM quality, with emphasis on the IgA, LTF, LYZ and BSSL levels and bioactivity by comparing pasteurization methods that follow the same temperature regime.

## Materials and Methods

### Milk Samples

**Figure 1:**
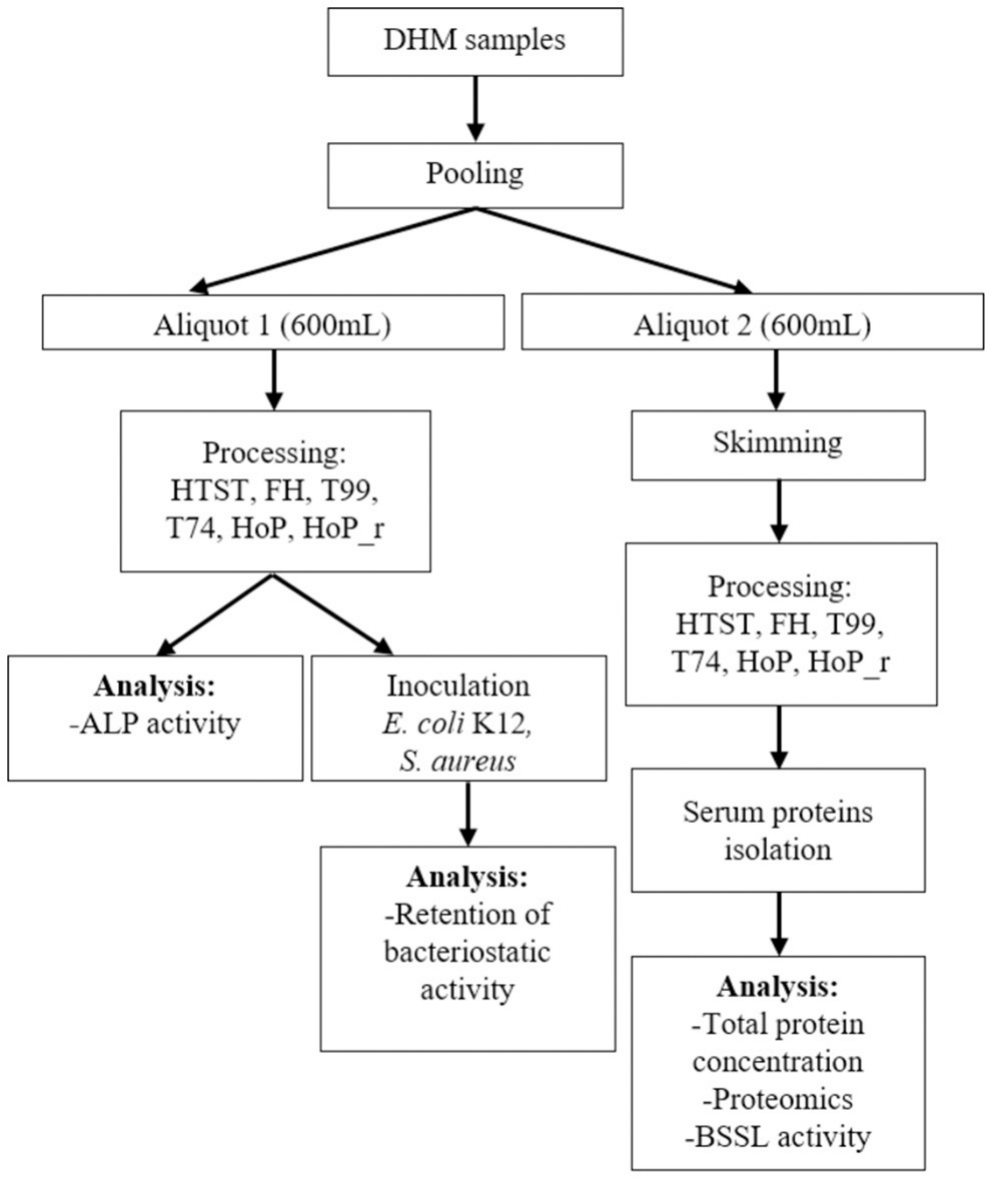
Schematic workflow indicating the experimental approach used. HTST, FH, T99, T74, HoP and HoP_r stand for high-temperature short-time, flash heating, high-temperature short-time with a thermomixer preheated at 99°C, high-temperature short-time with a thermomixer preheated at 74°C, holder pasteurization and holder pasteurization with rapid heating up-times, respectively. For each heating method, DHM treatment was done in biological duplicate. Each sample was then aliquoted for the subsequent analyses, which were performed in technical duplicate.

DHM samples were collected from the Dutch Human Milk Bank (located at Amsterdam UMC, Amsterdam, The Netherlands). Written informed consent was received from all donors before recruitment. Donor screening, milk expression and collection was conducted as per standardized protocols that adhere to internationally published guidelines (9). The samples were expressed with a breast pump and were subsequently collected in disposable bisphenol A-free bottles (Sterifeed, Medicare Colgate Ltd, Devon, England). The samples were immediately placed in a freezer at −20°C and were transported to the HMB, at a temperature of −20°C (21). All samples were placed in a refrigerator (4°C) overnight, before analysis. Next, milk samples from four different donors were combined into a pool, to ensure sufficient DHM amounts for all analysis. This pool was then divided into two aliquots (600 mL); one that was centrifuged at 6,500 × g for 30 min at 4°C (with rotor 16.250, Avanti Centrifuge J-26 XP, Beckman Coulter, USA) to remove the fat, and one that remained unskimmed. Then, both the unskimmed and the skimmed samples underwent heat treatment (with the methods mentioned in section 2.2), except for the untreated control samples. Skimming before heat treatment was done for the assays focusing on milk serum proteins, to avoid interference from the milk fat globule membrane. After treatment, all samples were cooled in an stationary ice-water bath and stored at −20°C. Figure 1 illustrates the experimental approach used in this study. The heating procedures were performed as two independent experiments (biological replicates).

### Heat Treatments

To compare the effects of processing times and temperatures on the DHM bioactive proteins, six pasteurization methods with different heating profiles were conducted; (1) HoP, (2) HoP with rapid heating up-times (HoP_r), (3) HTST, (4) flash heating (FH), (5) HTST with a thermomixer preheated at 99°C (T99), and (6) HTST with a thermomixer preheated at 74°C (T74). An overview of the different time-temperature profiles is shown as Supplementary Material (Supplementary Figures 1, 2).

#### Holder Pasteurization

A 130 mL single-use polypropylene bottle (Beldico SA, Marche-en-Famenne, BE) was filled with 100 mL of DHM and was pasteurized at 62.5°C for 30 min, in a shaking water bath (SW22, Julabo GmbH, DE) at 150 rpm. To achieve a rapid cooling phase, the sample was placed in an ice-water bath after treatment, until reaching a core temperature of 4°C. The temperature was recorded by a temperature data logger RS PRO 1,384 (RS Components B.V., The Netherlands). Three thermocouples were placed to monitor the temperature of the milk and of the two baths, during the whole process.

#### Holder Pasteurization With Rapid Heating Up-Times

The HTST system described in section High-Temperature Short-Time was used to achieve rapid heating up to the pasteurization temperature (62.5°C). The milk (50 mL) was pumped to the heating section and once its temperature reached 62.5°C, it was transferred in a shaking water bath for 30 min, as described in Holder Pasteurization.

#### High-Temperature Short-Time

**Figure 2:**
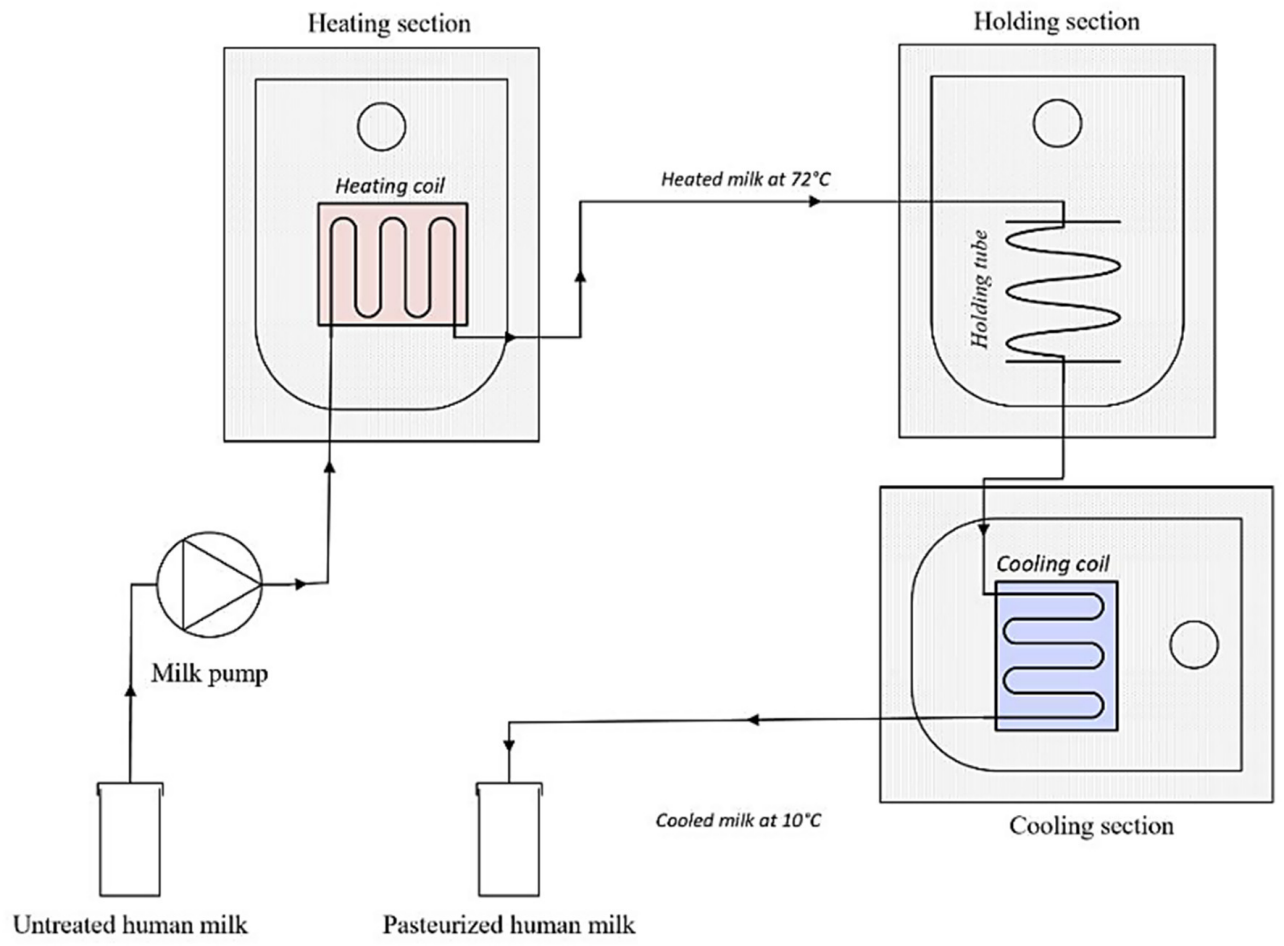
Schematic representation of the HTST system set-up.

A laboratory scale pasteurizer was built to simulate continuous HTST pasteurization. The system included a peristaltic pump (Watson Marlow 505S, Hudson, MA, USA), as well as a heating, a holding, and a cooling section (Figure 2). All sections were connected to an RS PRO 1,384 temperature data logger. The pump, which ran at a speed of 35 rpm, was connected with a plastic tube (ø 4 mm) to a copper heating coil (810 mm, ø 4 mm), that was fully submerged into a water bath (heating section). A thermocouple was placed at the end of the coil to determine whether the milk (50 mL) leaving the heating section indeed reached a temperature of 72°C. The coil was then connected to a plastic tube (ø 4 mm) that remained submerged into a second water bath (holding section, 15 sec). A second thermocouple was placed at the end of the holding section. The milk passed then through a copper coil (1,395 mm, ø 4 mm) which was submerged in an ice-water bath (2°C). Finally, a third thermocouple was linked with the coil to monitor the temperature at the end of the cooling phase. The cooled milk was then dispensed into a sterile bottle (Beldico SA, Marche-en-Famenne, BE).

#### Flash Heating

The set up used for this treatment was based on previous reports (22, 23). A 250 mL Duran bottle (GL 45, DWK Life Sciences GmbH, DE) was filled with 100 mL of DHM and was placed into an aluminum pan (2 L) containing 450 mL of water. The water and the submerged DHM bottle were heated simultaneously using an electric hot plate, until the water reached 100°C. The bottle was removed once the milk's temperature reached 71.5°C and was transferred into an ice-water bath (2°C). The temperatures of both milk and water were recorded by a temperature data logger (RS PRO 1,384).

#### Thermomixer Preheated at 99°C and 74°C

A shaking thermomixer (12 ml block, Eppendorf Thermomixer R Mixer, Hamburg, DE) was preheated to either 99°C or 74°C. Eight 12 mL Greiner tubes (Greiner Bio-One International GmbH, DE), each filled with 10 mL of DHM, were placed in the thermomixer once the desired temperature was reached (99°C or 74°C). The samples were shaken during the whole treatment (800 rpm), while the temperature was monitored with a temperature data logger. When the core temperature of the milk reached 72°C, the samples were removed from the thermomixer and after 15 s, they were then immediately transferred in an ice-water bath (2°C) for a rapid cooling down.

### Native Milk Serum and Total Protein Content

To obtain the native serum protein fraction from the skimmed DHM samples, caseins and denatured proteins were precipitated by acidifying the samples with HCl (1 mol/L) under stirring, until a pH of 4.6 was reached (24). After leaving it for 30 min at 4°C to equilibrate, the samples were ultracentrifuged for 90 min at 100,000 × g and at 30°C (Optima L-80, Beckman Coulter, USA). Next, the supernatant with the native serum protein fraction was collected and the pellet containing micellar casein and denatured proteins was discarded. Finally, total protein content was determined with the bicinchoninic acid (BCA) assay kit (Thermo Fisher Scientific, USA), as specified in the manufacturer's instructions.

### Proteomics by Liquid Chromatography With Tandem Mass Spectrometry (LC-MS/MS)

#### Filter Aided Sample Preparation for Proteomics

Before LC-MS/MS measurements, the samples were prepared with the FASP method, as previously described (25). Briefly, after the samples (1.0 μg/μL) were reduced with 15 mM dithiothreitol, they were first diluted by urea (8 M) in 100 mM Tris/HCl (0.1 M, pH 8.0) and then alkylated with the addition of 20 mM of acrylamide (0.2 M). Next, 100 μL of the alkylated samples were transferred to a Pall 3 K omega filter (10–20 kDa cut off, OD003C34, Pall corporation, Port Washington NY, USA) and were centrifuged for 30 min at 16,900 × g. The samples were then washed with 50 mmol/L NH_4_HCO_3_ (ABC) and were centrifuged again at the same conditions. The filter units were placed into new low-binding tubes (2 mL) and the samples were subjected to overnight digestion by the addition of 100 μL trypsin in NH_4_HCO_3_ solution (5 ng/μL). Next, a centrifugion step at 16,900 × g for 30 min followed, and another one at the same conditions after the addition of 100 μL 1 mL/L HCOOH in water on top of the filter unit. Finally, 3 μL of 10% trifluoroacetic acid was added to the filtrate to adjust sample pH to around 3. Before injection into the LC-MS/MS system, all samples were stored at −20°C.

#### LC-MS/MS Proteomics

All analyses were carried out by the department of Biochemistry at Wageningen University and Research. The parameters used were the same as previously reported (25). The samples were directly injected on a 0.10^*^250 mm ReproSil-Pur 120 C18-AQ 1.9 μm beads analytical column prepared in house, at 800 bar. Elution of the peptides was done at a flow of 0.5 μL/min, using an acetonitrile gradient (9–34% acetonitrile in water with 1 ml/L formic acid in 50 min). The eluent was then ejected trough the tip of a needle with an electrospray potential of 3.5 kV. Full scan FTMS in positive mode between m/z 380 and 1,400 were measured using Q Exactive HF-X mass spectrometer (Thermo Electron, San Jose, CA, USA). MS/MS scans of the twenty most abundant multiply charged peaks, were measured in data-dependent mode. MaxQuant software (v1.6.3.4) was used to analyze the obtained MS data, against the Uniprot human protein database and a database containing the sequences of common contaminants (26). Protein modifications were set as propionamide (C) (fixed) and oxidation (M) (variable), while enzyme specificity was set for trypsin and a maximum of two missed cleavages, 20 ppm peptide tolerance first search, 4.5 ppm main search and 20 ppm MS/MS fragment match tolerance. Requirement for further analysis was the protein identification by a minimum of two peptides that had at least one unique and one unmodified peptide. Proteins detected in less than half of the samples as well as keratins and trypsin were removed from the final list of identified proteins.

### BSSL Activity

The assay used to determine BSSL activity was based on a previously published method, with minor modification (27). DHM lipase activity is determined fluorometrically through the utilization of two synthetic substrates; 4-methylumbelliferyl butyrate (4-MUB) and 4-methylumbelliferyl laurate (4-MUL). Defatted DHM samples were preincubated at 40°C for 3 min, under 800 rpm, in a ThermoMixer (SmartBlock 1.5 ml, Eppendorf, Hamburg, DE). A stop solution of GuHCl (8 M) and HCl (1 M) in water was then used to stop the conversion of the added substrate and a neutralizing solution with Bis-tris (1M), NaOH (0.85 M) and EDTA (0.25 M) in water was added next to clarify the samples. The fluorescence released was measured by using a fluorimeter (SpectraMax ID3, Molecular Devices, San Jose, CA, USA) at an excitation of 355 nm and an emission of 460 nm.

### Alkaline Phosphatase

The method used for the detection of ALP was according to an international standard protocol (ISO/TS 6090|IDF/RM 82A:2004). Finally, ALP activity was measured in a p-nitrophenol calorimeter (Lovibond APTW/7, Tintometer GmbH, Dortmund, DE).

### Bacteriostatic Properties

In order to assess the effect of the different methods on the functionality of the three major HM antimicrobial proteins (IgA, LTF, LYZ), we evaluated the growth rate of two bacterial strains known to be inhibited by these proteins. Fresh cultures of *Escherichia coli K12* (DSM 498, DSMZ, Braunschweig, Germany) and *Staphylococcus aureus* (ATCC 6,538, American Type Culture Collection, Manassas, USA) were prepared from frozen stocks in nutrient broth overnight at 37°C (CM0001, Thermo Fisher Scientific, Massachusetts, USA). Bacterial pellets were obtained after a centrifugation step of 10 min at 4,000 × g (Microcentrifuge 5890R, Eppendorf, Hamburg, Germany) and were subsequently dissolved in peptone physiological salt solutions (PFZ; Tritium Microbiology, The Netherlands). Optical density was determined by using a spectrophotometer (Cary 50 UV–Visible Spectrophotometer, Agilent Technologies, USA). Next, *E. coli* and *S. aureus* cultures were inoculated into untreated samples and samples that were first subjected to heat treatment with the different methods, to a concentration of ~10^3^ colony forming units (CFU)/mL. DHM samples inoculated with *E. coli* were incubated at 37°C for 2 h and the samples inoculated with *S. aureus* for 4 h, at the same temperature. All samples were plated in duplicate onto selective media; violet red bile glucose agar for *E. coli* (CM0107B, Thermo Fisher Scientific, Massachusetts, USA) and mannitol salt agar for *S. aureus* (CM0085B, Thermo Fisher Scientific, Massachusetts, USA) and were then incubated overnight at 37°C. Bacterial counts were determined by colony counting (CFU/mL) while the growth rare per hour was measured as $\ln\left(\frac{N_{t}}{N_{0}}\right)/t$, were N_t_ = bacterial counts after either 2 h or 4 h of incubation, N_0_ = bacterial counts immediately after incubation and t = incubation time.

### Data Analysis

GraphPad Prism software 8.0 (GraphPad Inc., La Jolla, CA) was used for data analysis and visualization. The effects of the different treatments were compared by ANOVA and Tukey's HSD for *post-hoc* tests. Protein retentions (%) were determined after dividing the concentrations after treatment by the concentration of untreated samples, multiplied by 100. The intensity based absolute quantification (iBAQ) values obtained with MaxQuant, were analyzed in Perseus software (v.1.6.2.1, Martinsreid, Germany). The iBAQ values are considered as suitable indicators for absolute protein concentrations, as the values refer to the sum of all peptide intensities divided by the number of theoretically generated tryptic peptides (28). Perseus was used to estimate significant differences in the protein pattern after treatment, by Student's *t*-tests with permutation-based false discovery rate (FDR) correction. The correlation between the levels of IgA, LTF and LYZ retained and the bacterial growth rate was also determined, by creating a correlation matrix with R version 3.4.0 (29). A *p*-value < 0.05 was used to indicate significant differences among the compared groups. The analyses were performed in duplicate for each sample and all data are shown as mean ± standard deviation of two independent experiments.

## Results

### Temperature Profiles

The temperature profiles of the different treatments were broken down into three sections: the heating up time to the pasteurization temperature (referred to as heating up time), the time that DHM was held at this temperature, and the time required for DHM to cool down to 4°C (Table 1). The time DHM spent above 55°C was used as an indicator of the thermally induced protein denaturation that usually occurs above this temperature (30). During HoP, the samples were exposed to temperatures above 55°C for about 44 min, which was the longest exposure observed among the different treatments. In contrast, HTST-treated DHM was exposed above this temperature for only 33 sec.

**Table 1:** Time-temperature profiles of the six processing methods.

**Processing methods**	**Volume processed (mL)**	**Time above 55°C (min)**	**Heating up time (min)**	**Holding time (min)**	**Cooling down time (min)**
HTST	50	0.33	0.12	0.25	0.18
FH	100	5	9	0.25	7.5
T99	80	7.3	11	0.25	3.3
T74	80	32	40	0.25	2.3
HoP_r	50	31.25	0.10	30	16
HoP	100	44	27	30	16

*HTST, FH, T99, T74, HoP and HoP_r stand for high-temperature short-time, flash heating, high-temperature short-time with a thermomixer preheated at 99°C, high-temperature short-time with a thermomixer preheated at 74°C, holder pasteurization and holder pasteurization with rapid heating up-times, respectively*.

### Native Milk Serum Protein Concentration and Quantitative Analysis of the Milk Serum Proteome

**Figure 3:**
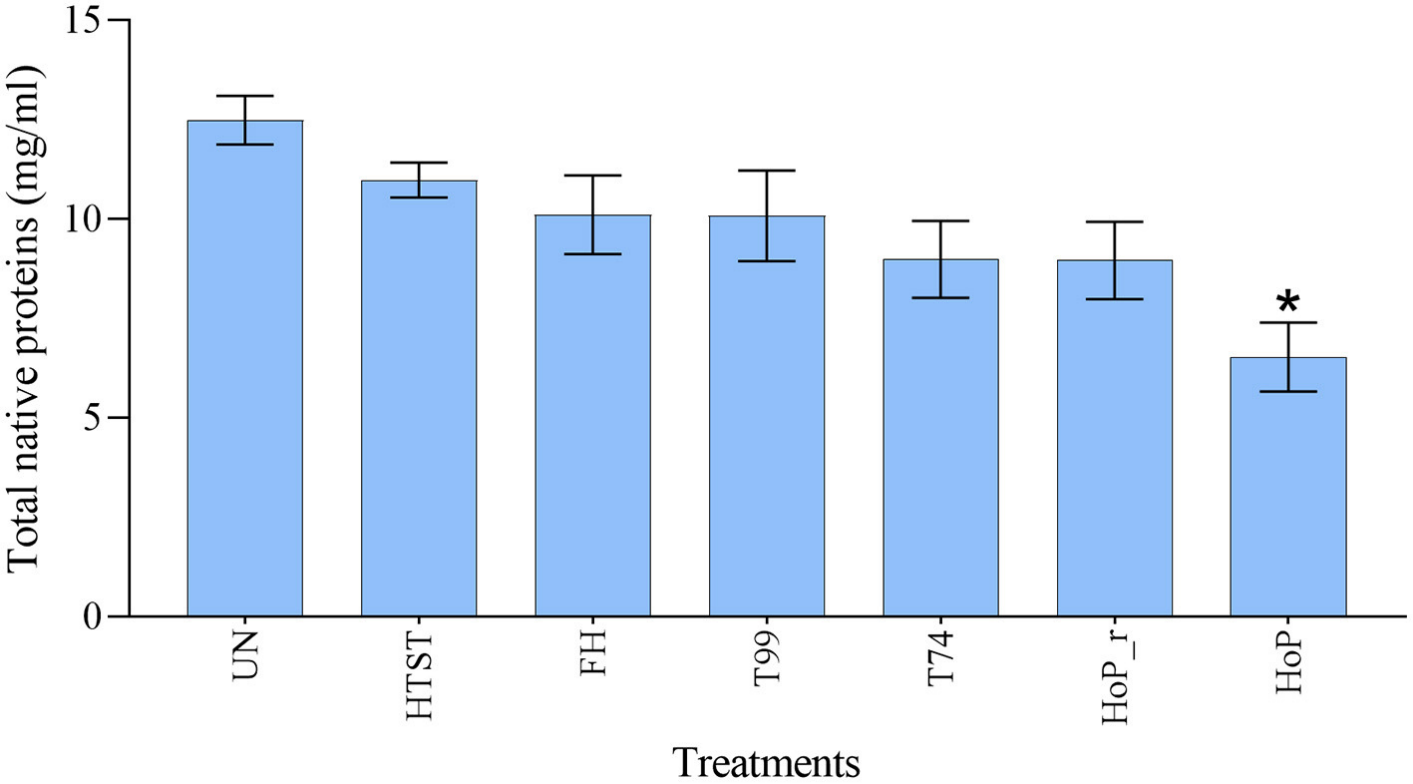
Total native milk serum protein concentration (mg/ml) of untreated and the differently heat-treated DHM samples. The analyses were performed in duplicate for each sample and all values are presented as mean ± standard deviation of two independent experiments. *Expresses statistically significant difference to untreated samples (*p* < 0.05). UN, HTST, FH, T99, T74, HoP and HoP_r stand for untreated DHM, and DHM treated with high-temperature short-time, flash heating, high-temperature short-time with a thermomixer preheated at 99°C, high-temperature short-time with a thermomixer preheated at 74°C, holder pasteurization and holder pasteurization with rapid heating up-times, respectively.

**Figure 4:**
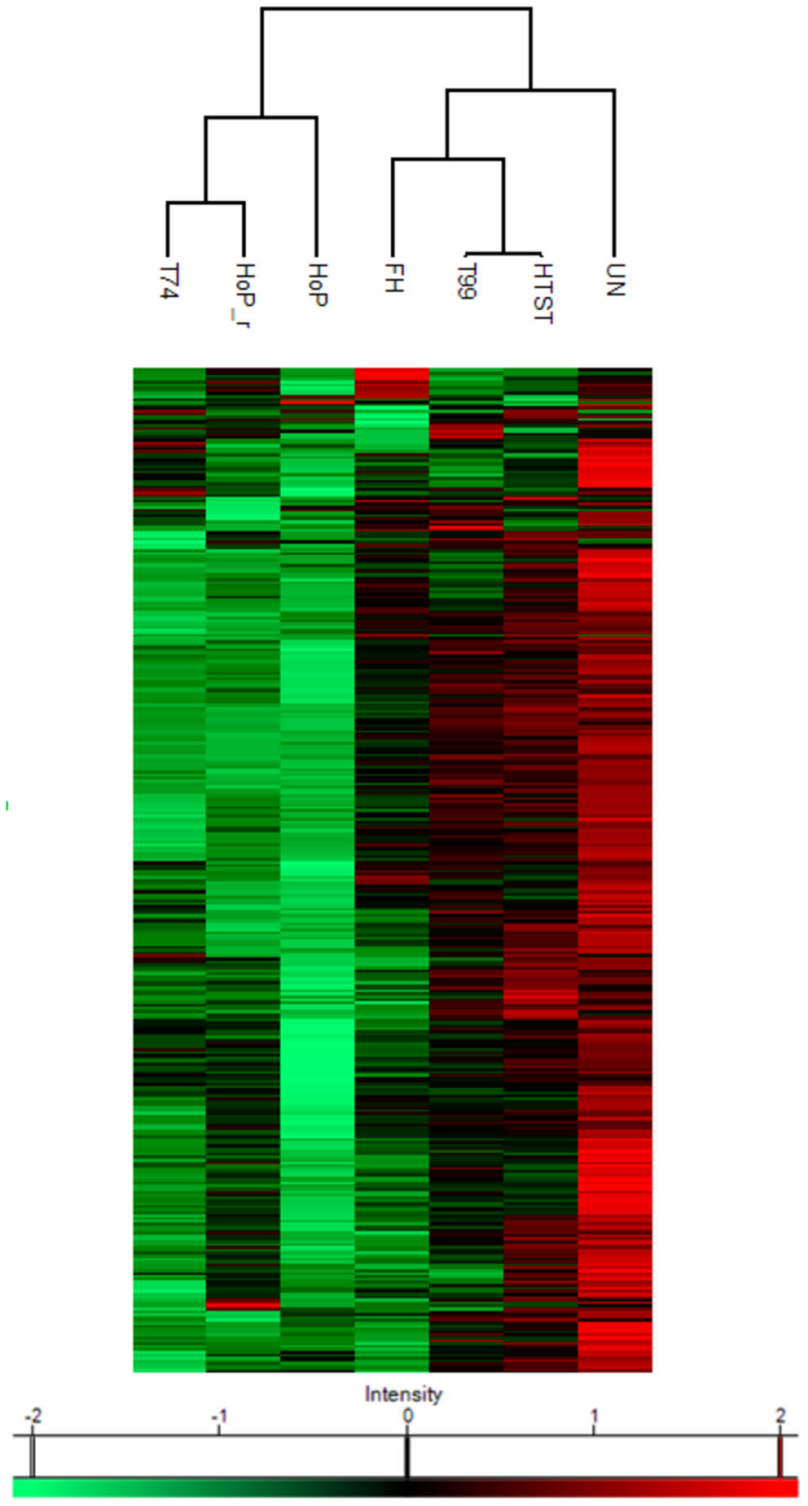
Heat map indicating differences in protein profile of the differentially heated DHM samples. The color scale is based on z-score normalized iBAQ values and each row represents individual proteins. Hierarchical clustering was performed using a Euclidean distance metric. The analyses were performed in duplicate for each sample and all values are presented as mean ± standard deviation of two independent experiments UN, HTST, FH, T99, T74, HoP and HoP_r stand for untreated DHM, and DHM treated with high-temperature short-time, flash heating, high-temperature short-time with a thermomixer preheated at 99°C, high-temperature short-time with a thermomixer preheated at 74°C, holder pasteurization and holder pasteurization with rapid heating up-times, respectively.

A combination of acidification and ultracentrifugation was applied to specifically isolate the native milk serum proteins. The total native milk serum protein concentration of the untreated and the treated samples is shown in Figure 3. Of the treatments tested in this study, only HoP caused a significant decrease in native protein concentration (*p* < 0.05), when compared to the untreated samples. In addition to the total native protein content, the native protein profile was assessed as well by LC-MS/MS. The impact of the different heat treatments on the DHM native protein profile was then visualized by a clustered heat map of the obtained iBAQ values (Figure 4). According to the clustering pattern (Figure 4), the native protein profile of the samples that were the longest exposed to temperatures >55°C (samples treated with T74, HoP or HoP_r) differed the most from the protein profile of the untreated samples.

### IgA, LTF and LYZ Levels After Processing

**Figure 5:**
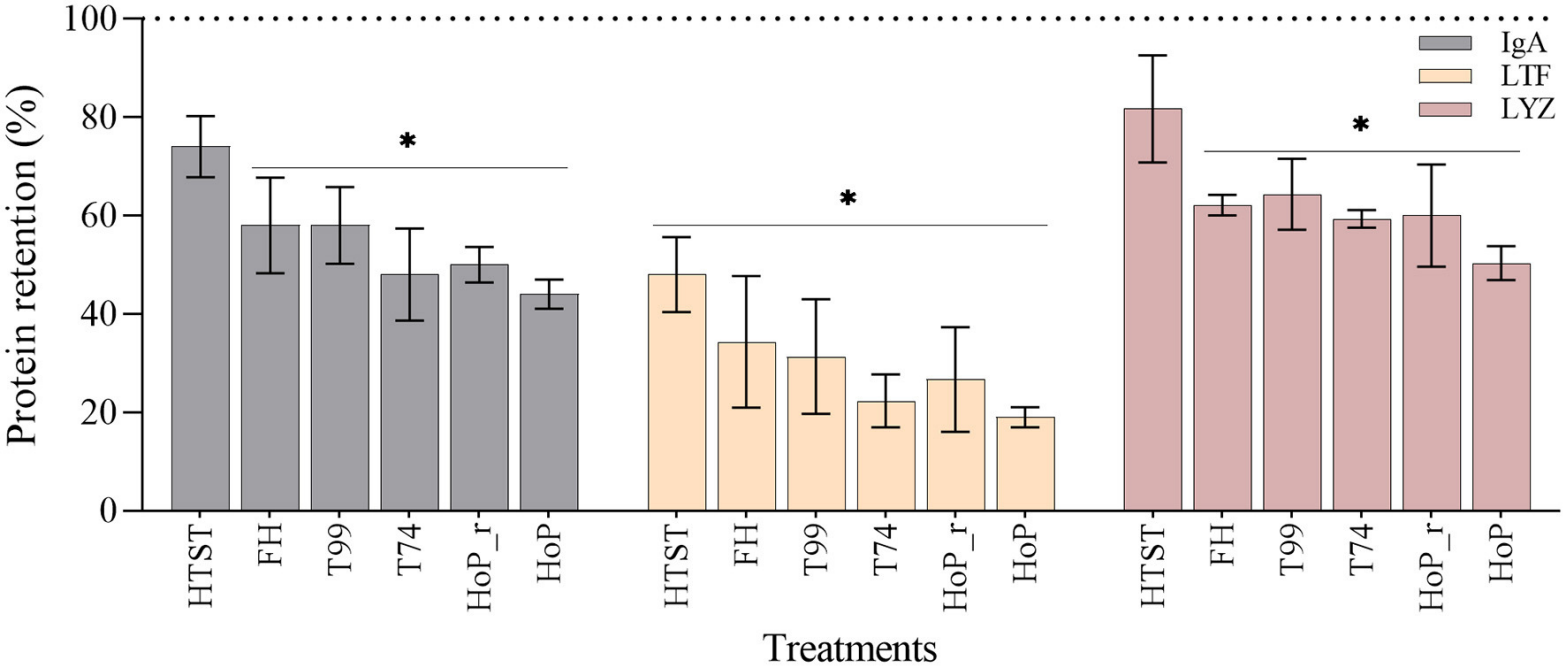
Effect of HTST, FH, T99, HoP_r, T74 and HoP on the IgA, LTF and LYZ content. Protein retention (%) was calculated based on the iBAQ intensities obtained by LC-MS/MS. The dotted line represents the untreated values (100%). The analyses were performed in duplicate for each sample and all values are presented as mean ± standard deviation of two independent experiments. *Expresses statistically significant differences to untreated samples (*p* < 0.05). UN, HTST, FH, T99, T74, HoP and HoP_r stand for untreated DHM, and DHM treated with high-temperature short-time, flash heating, high-temperature short-time with a thermomixer preheated at 99°C, high-temperature short-time with a thermomixer preheated at 74°C, holder pasteurization and holder pasteurization with rapid heating up-times, respectively.

Overall, the retentions of the three proteins showed a decreasing tendency with increasing exposure time above 55°C. Compared to untreated DHM, FH, T99, HoP_r, T74 and HoP significantly reduced the IgA, LTF and LYZ levels (*p* < 0.05), with average retention rates between 19 and 64% (Figure 5). HoP preserved IgA, LTF and LYZ levels the least, but HoP with rapid heating up times was shown to improve their retention, although the differences observed between the two methods were non-significant (mean ± SD retention rates of IgA, LTF and LYZ after HoP_r and HoP; 50 ± 5% vs. 44 ± 4%, 26 ± 18% vs. 19 ± 4%, 60 ± 18% vs. 50 ± 6%, respectively, *p* > 0.05). HTST, the treatment with the shortest processing times, was found to better retain the levels of the three proteins; the concentrations of IgA and LYZ were not significantly different from those of untreated samples (74 ± 9 and 82 ± 19%, respectively) but the concentration of LTF was significantly reduced (48 ± 13% of LTF was retained, *p* < 0.05). However, the concentration of LTF after HTST was significantly higher than after HoP and T74 (*p* < 0.05). Our data also showed that the IgA levels were significantly higher after HTST than after HoP (*p* < 0.05), but for LYZ, no significant differences were observed between the two treatments.

### BSSL Level and Activity After Processing

**Figure 6:**
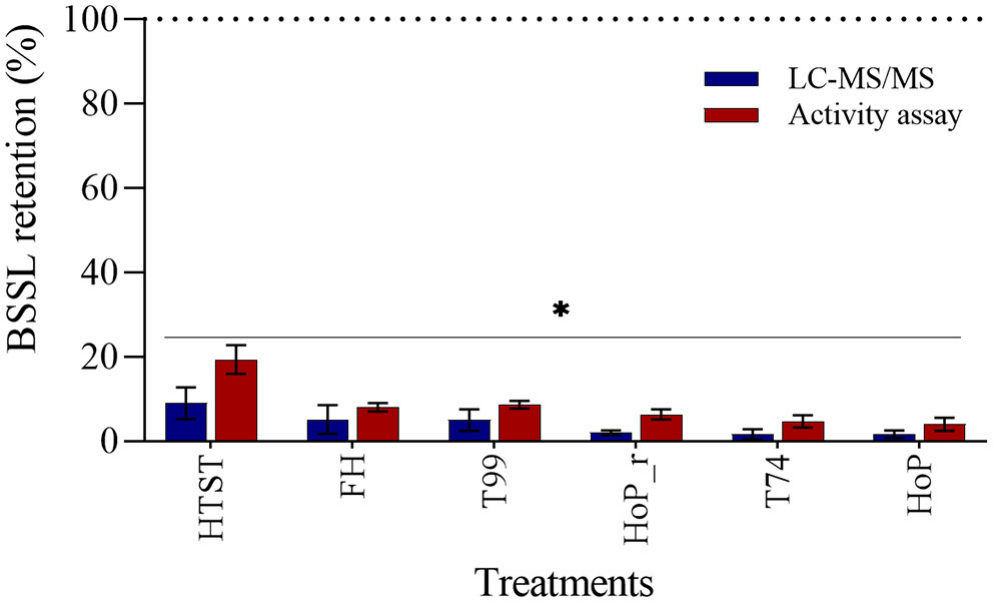
Effect of HTST, FH, T99, HoP_r, T74 and HoP on BSSL levels. BSSL retention (%) was calculated based on the iBAQ intensities and a lipase activity assay. Untreated values were set at 100% (dotted line). The analyses were performed in duplicate for each sample and all values are presented as mean ± standard deviation of two independent experiments. *Expresses statistically significant differences to untreated samples (*p* < 0.05). UN, HTST, FH, T99, T74, HoP and HoP_r stand for untreated DHM, and DHM treated with high-temperature short-time, flash heating, high-temperature short-time with a thermomixer preheated at 99°C, high-temperature short-time with a thermomixer preheated at 74°C, holder pasteurization and holder pasteurization with rapid heating up-times, respectively.

The effects of the different heat treatments on BSSL level and activity were determined by means of LC-MS/MS and an activity assay. All treatments caused a major reduction on the enzyme's level and activity, with respect to untreated DHM (*p* < 0.05, Figure 6). BSSL was affected the most by HoP (LC-MS/MS, 2% and activity assay, 4%), but the values obtained after FH, T99, HoP_r and T74 were comparable to those of HoP (*p* > 0.05). HTST retained significantly higher BSSL level and activity than the other treatments (LC-MS/MS, 9% and activity assay, 19%, *p* < 0.05).

### ALP Activity After Processing

ALP is a very heat-sensitive enzyme and it is expected to be completely inactivated when the pasteurization is adequate (19). Untreated DHM samples exhibited a mean ALP activity of 0.257 ± 0.049 U/ml, whereas all heat-treated samples were below the detection limit.

### Retention of Bacteriostatic Properties After Processing

**Figure 7:**
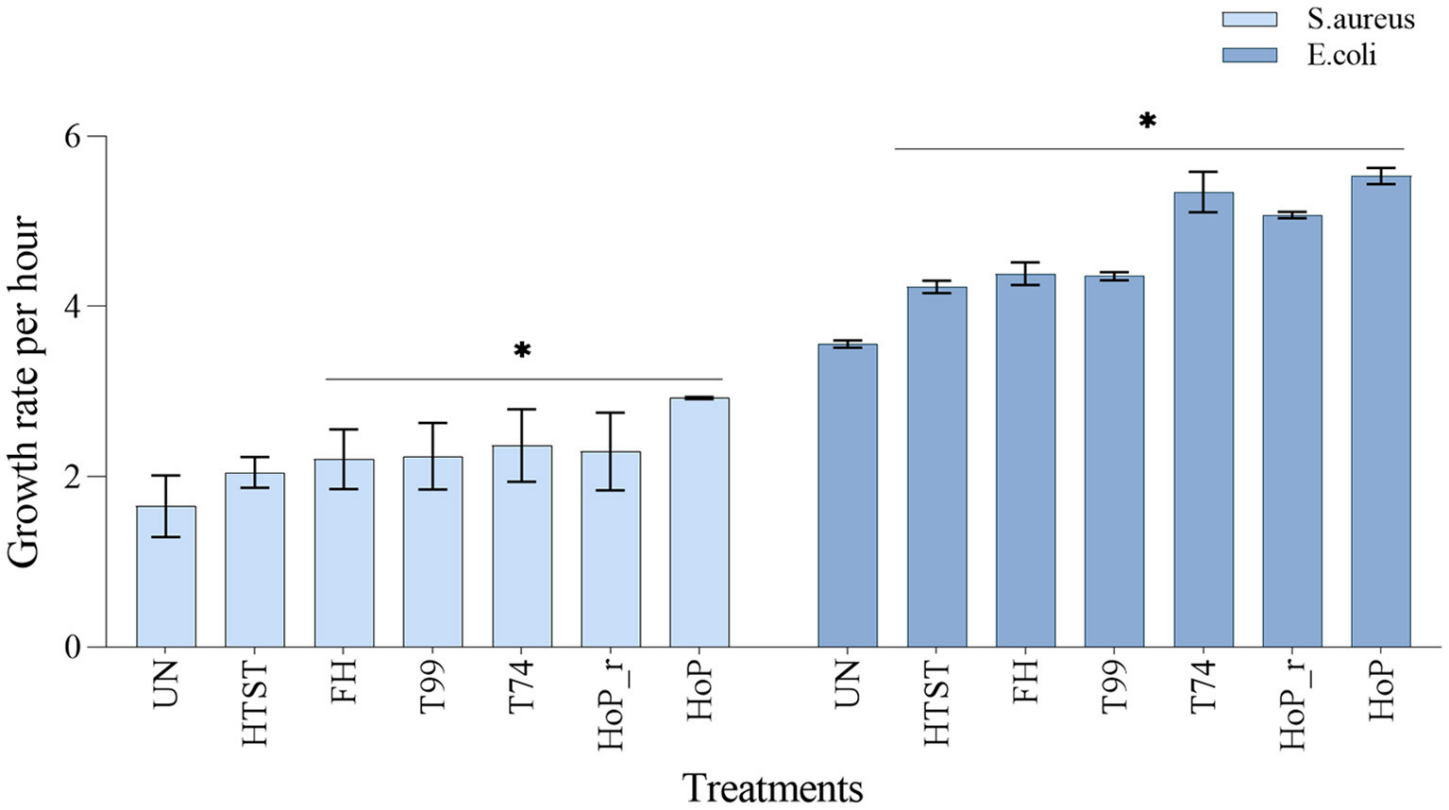
Growth rate per hour of *S. aureus* and *E. coli* in untreated DHM samples and after HTST, FH, T99, HoP_r, T74 and HoP. The analyses were performed in duplicate for each sample and all values are presented as mean ± standard deviation of two independent experiments. *Expresses statistically significant differences to untreated samples (*p* < 0.05). UN, HTST, FH, T99, T74, HoP and HoP_r stand for untreated DHM, and DHM treated with high-temperature short-time, flash heating, high-temperature short-time with a thermomixer preheated at 99°C, high-temperature short-time with a thermomixer preheated at 74°C, holder pasteurization and holder pasteurization with rapid heating up-times, respectively.

To assess the impact of the different heat treatments on the DHM bacteriostatic capacity, the growth rates of *S. aureus* and *E. coli* were evaluated, in untreated and in heat-treated samples (Figure 7). The lowest growth rate for both strains was documented in untreated DHM (1.7 ± 0.36 and 3.6 ± 0.04-fold per hour, for *S. aureus* and *E. coli*, respectively), which indicates that untreated DHM samples exhibited the highest bacteriostatic capacity among all samples (*p* < 0.05). In contrast, HoP caused the highest reduction in bacteriostatic capacity (*S. aureus* and *E. coli* growth rate, 2.9 ± 0.01 and 5.53 ± 0.09-fold per hour, respectively, *p* < 0.05). Compared to the untreated samples, HTST resulted in a comparable *S. aureus* growth rate (2.0 ± 0.17-fold per hour, *p* > 0.05) but the *E. coli* growth rate increased significantly after this treatment (4.2 ± 0.07-fold per hour, *p* < 0.05). Similarly, the bacterial growth rate was significantly increased after FH, T99, HoP_r and T74 (*p* < 0.05). When compared to HoP, *S. aureus* growth was significantly lower after HTST, FH, T99, HoP_r and T74, but when *E. coli* growth was assessed, that was the case only for the samples after HTST, FH and T99 (*p* < 0.05).

**Figure 8:**
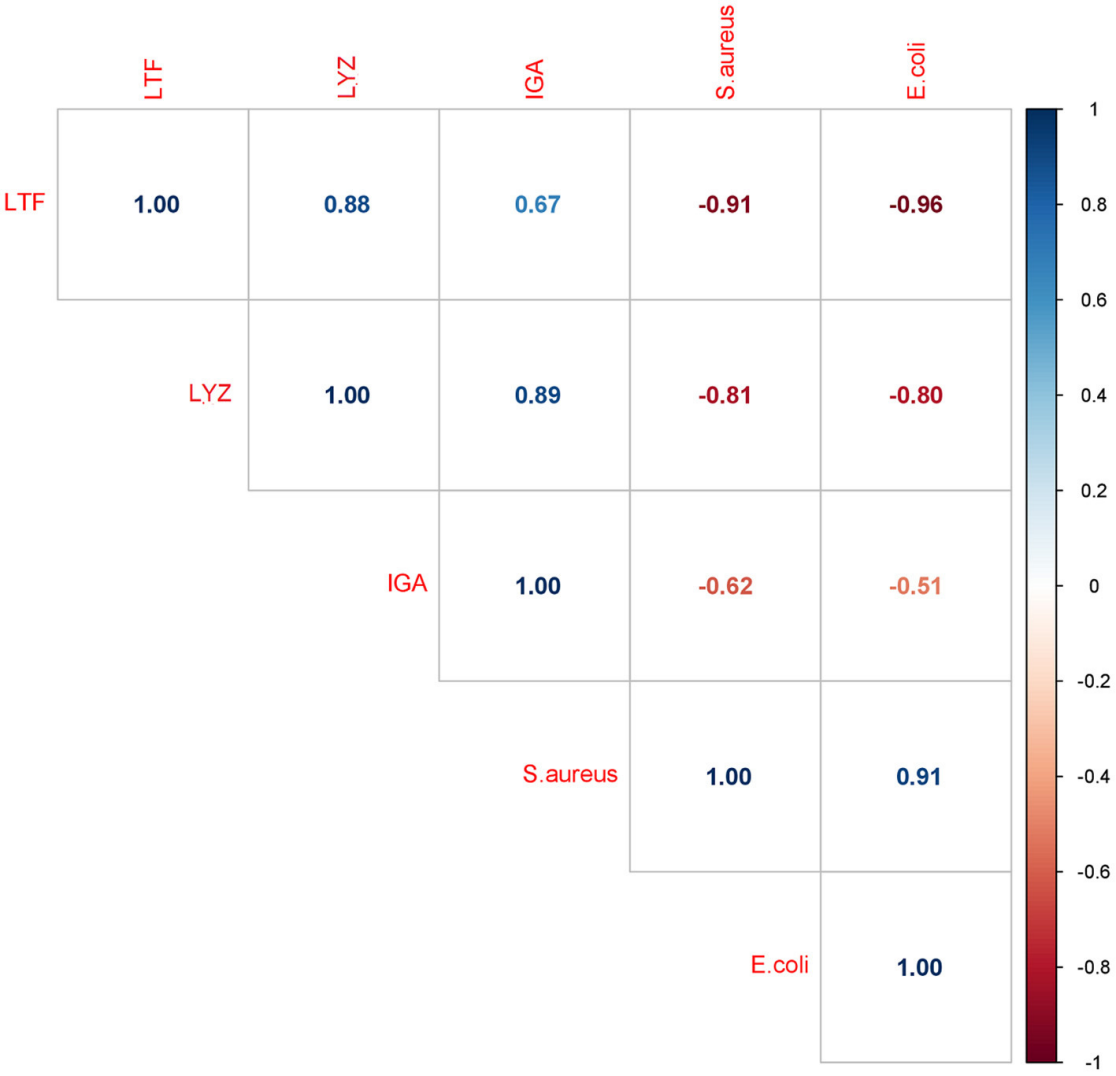
Correlation matrix of bacterial growth rates and IgA, LTF and LYZ iBAQ intensities. Each box includes a Pearson correlation coefficient (*r* value).

Considering that the IgA, LTF and LYZ levels decreased while the *S. aureus* and *E. coli* growth rate increased, a negative correlation between bacterial growth rate and the retention of the three major antimicrobial proteins is expected, as was indeed found (Figure 8). The strongest negative correlation was observed between bacterial growth rate and the levels of LTF and LYZ (LTF; *r* = −0.91 and *r* = −0.96, LYZ; *r* = −0.81 and *r* = −0.80, for *S.aureus* and *E.coli* respectively, *p* < 0.05). The correlation between IgA levels and bacterial growth rate was weaker but still significant (*S.aureus, r* = −0.62 and *E.coli, r* = −0.51, *p* < 0.05).

## Discussion

The current study illustrates that both shorter heating up times and shorter duration of pasteurization at a higher temperature preserved the levels and bioactivity of key DHM bioactive components better than HoP. In addition, the ALP assay showed that all tested time-temperature combinations resulted in the complete inactivation of the enzyme, indicating that sufficient heat load was applied.

### Effects of Processing Times and Temperatures on IgA, LTF and LYZ Levels and Activity

Our results confirmed the major impact of HoP on the DHM protein profile (16). HTST, FH and T99, the treatments with the shortest processing times, seemed to better preserve the DHM protein profile when compared to HoP_r, T74 and HoP. Among the methods tested, HTST showed the least reduction in the levels of the studied bioactive proteins, while HoP showed the highest reduction in their levels. More specifically, IgA and LYZ levels after HTST were not significantly different to those of untreated samples, while LTF was significantly reduced after all thermal treatments. These differences were to be expected as DHM was exposed above 55°C the shortest after HTST (0.33 min) and the longest after HoP (44 min).

Our findings on the advantages of HTST over HoP in the retention of key DHM bioactive components are in line with the literature (14, 16, 19, 31–33). However, considerable variations in the losses of IgA, LTF and LYZ after HTST are reported; 0–60% of IgA and 0–85% of LTF, while for LYZ, losses up to 40% and increases up to 28% were reported (32, 34–37). Several reasons may explain these variations, including the fact that the extent of protein denaturation depends both on their physicochemical characteristics and the nature of the thermal treatment (38). The different HTST devices (e.g., laboratory or industrial heat exchangers, other benchtop devices or by immersion in thermostatically controlled water baths in bulk processes), the differences in holding times (5–25 s) and temperatures (62–87°C) as well as differences in the methods of analysis in these studies (e.g., ELISAs, radial immunodiffusion assays, enzymatic activity assays, mass spectrometric methods) may have also contributed to different protein retentions documented (15–19, 32, 33, 35, 36, 39).

In respect to the impact of the different heat treatment parameters on the studied DHM bioactive components, among the treatments following the same temperature regime as HTST (15 s at 72°C), T74 caused the highest protein loss. This could be attributed to the longer heating up time documented during T74 (40 min), which was the result of the small heat exchanging surface area and the small temperature differences between the heating medium and the desired pasteurization temperature. Similarly, HoP_r performed slightly better, although not significantly, than HoP. The possible explanation could be that HoP and HoP_r both follow the same holding regime (30 min at 62.5°C), but the heating up time is much shorter for HoP_r (0.10 min) than during HoP (27 min). When evaluating the performance of the two treatments with the shortest heating up times, HTST and HoP_r, it was clear that the considerably longer holding time during HoP_r was the reason of the higher protein damage caused. These observations suggest that the combination of processing parameters that leads to a prolonged exposure above temperatures of 55°C is of crucial importance for the retention of the three studied proteins.

In accordance with our results, Buffin et al. also showed that an optimized HoP, with a mean plateau temperature of 1.5°C lower and duration of 11 min shorter than HoP, preserved higher amounts of IgA, LTF and LYZ (40). In addition, Escuder-Vieco et al. found a 30% decrease in IgA concentration, regardless of the temperature-time combination used for HTST (5–25 s at 70–75°C), while Mayayo et al. showed that the largest reductions in the IgA and LTF levels during HoP were documented during the first 5 min of treatment (45 and 70% for IgA and LTF, respectively), with the remaining 25 min of treatment causing <10% reductions (35, 41, 42). For LYZ, studies showed contradictory results in the effect of thermal treatments due to its stable structure, which may be explained by the different analytical approaches used to measure its activity (17, 35, 36, 43, 44).

When the effect of the different heat treatment parameters on the DHM bacteriostatic capacity was assessed, a decrease of the bacteriostatic capacity with increasing exposure times above 55°C was observed. Furthermore, the correlations observed between IgA, LTF and LYZ levels and the growth rate of *S. aureus* and *E. coli*, which are sensitive to these proteins, indicate that these proteins may have a significant role in retarding their growth. Of the treatments performed in the current study, HoP-treated DHM was found to exert the lowest bacteriostatic capacity, while the bacteriostatic capacity of HTST-treated DHM was significantly decreased against *E. coli* but unaffected against *S. aureus*. These results are in good agreement with the IgA, LTF and LYZ losses documented after these treatments. Especially for LTF, heat treatments have been shown to reduce its iron-binding capacity (45), which may have contributed to the significant increase in the *E. coli* growth rate in all heat-treated DHM samples. Other studies investigating the effect of heat treatment on the DHM bacteriostatic capacity, found a similar decrease after HoP (5, 46, 47). The heat-induced denaturation and aggregation during HoP could further explain the loss of protein functionality (13, 48). In contrast to our findings, Silvestre et al. reported a higher decrease in the DHM bacteriostatic capacity after pasteurization at 75°C for 15 s than after 63°C for 30 min (49). These differences may be attributed to the different pasteurization designs and the different bacterial strains used. Taken together, treatments with longer processing times, such as HoP, have a significantly larger impact on the DHM bacteriostatic capacity.

### Effects of Processing Times and Temperatures on BSSL Level and Activity

Since BSSL is a heat labile enzyme that starts inactivating at temperatures of 45°C (30), the great loss documented after all thermal treatments was to be expected. Wardell et al. showed that even a short exposure at 55°C can inactivate the enzyme, which explains the <20% retention that was documented after HTST. BSSL level and activity were almost completely abolished after HoP, as previously reported (10, 19, 50, 51). FH, T99, HoP_r and T74 affected BSSL in a similar manner, independently of the different heating up and holding times applied. When comparing HTST to HoP, the significantly higher BSSL activity detected after HTST, may be attributed to the considerably shorter DHM exposure time over 55°C (33 s vs. 44 min). Similar observations have been previously documented (19, 51). These findings suggest that non-thermal processing methods, such as ultraviolet-C irradiation or high-pressure processing, may offer substantially better results (50, 52).

## Conclusion

Heat treatments, such as HTST, with considerably shorter processing times than the currently recommended HoP, were found to improve the retention of key DHM bioactive components. Our findings suggest that both reduced heating up and holding times are an essential factor for pasteurization optimization, and lead to improved DHM quality. Since the recipients of pasteurized DHM are high-risk infants, these outcomes are of crucial importance. The pasteurization treatments used in this study are all assumed to result in a safe product based on the inactivation of alkaline phosphatase, but whether inactivation of spore-forming pathogens such as B. cereus is achieved with such treatments is yet unknown. Moreover, considering that all thermal treatments caused a major reduction in BSSL levels and activity, in LTF and in bacteriostatic capacity, future studies should additionally investigate the effects of non-thermal methods on these components.

## Data Availability Statement

The raw data supporting the conclusions of this article will be made available by the authors, without undue reservation.

## Author Contributions

EK performed the experimental work, analyzed the obtained data, and wrote the manuscript. BS, JvG, RvE, and KH contributed to the conception, the supervision of the study, and critically reviewed the manuscript. SB performed the LC-MS/MS measurements and critically reviewed the manuscript. All authors contributed to the article and approved the submitted version.

## Conflict of Interest

JvG is the founder and director of the Dutch National Human Milk Bank and member of the Dutch National Health Council. BS is as Science Director of Human Milk Research and Analytical Sciences an employee of Danone Nutricia Research, Utrecht, Netherlands. The remaining authors declare that the research was conducted in the absence of any commercial or financial relationships that could be construed as a potential conflict of interest.

## Publisher's Note

All claims expressed in this article are solely those of the authors and do not necessarily represent those of their affiliated organizations, or those of the publisher, the editors and the reviewers. Any product that may be evaluated in this article, or claim that may be made by its manufacturer, is not guaranteed or endorsed by the publisher.
