# Supplementary material for: High-Temperature Short-Time Preserves Human Milk's Bioactive Proteins and Their Function Better Than Pasteurization Techniques With Long Processing Times
Source: Front Pediatr. 2022 Jan 20;9:798609. doi: 10.3389/fped.2021.798609 (PMC8811466; doi:10.3389/fped.2021.798609)
Supplement: Supplementary file 1 [file Data_Sheet_1.docx]

**Supplementary material**

**Figure S1.** Time-temperature profiles of pasteurization methods at 72°C for 15 sec. HTST, FH, T99 and T74 stand for high-temperature short-time, flash heating, high-temperature short-time with a thermomixer preheated at 99°C and high-temperature short-time with a thermomixer preheated at 74°C, respectively.

**Figure S2.** Time-temperature profiles of pasteurization methods at 62.5°C for 30 min. HoP and HoP_r stand for holder pasteurization and holder pasteurization with rapid heating up-times, respectively.
